# Improving biomedical information retrieval by linear combinations of different query expansion techniques

**DOI:** 10.1186/s12859-016-1092-8

**Published:** 2016-07-25

**Authors:** Ahmed AbdoAziz Ahmed Abdulla, Hongfei Lin, Bo Xu, Santosh Kumar Banbhrani

**Affiliations:** School of Computer Science and Technology, Dalian University of Technology, No.2 Linggong Rd., Dalian, 116024 People’s Republic of China

**Keywords:** Query expansion, Biomedical information retrieval, Linear combination of query results

## Abstract

**Background:**

Biomedical literature retrieval is becoming increasingly complex, and there is a fundamental need for advanced information retrieval systems. Information Retrieval (IR) programs scour unstructured materials such as text documents in large reserves of data that are usually stored on computers. IR is related to the representation, storage, and organization of information items, as well as to access. In IR one of the main problems is to determine which documents are relevant and which are not to the user’s needs. Under the current regime, users cannot precisely construct queries in an accurate way to retrieve particular pieces of data from large reserves of data. Basic information retrieval systems are producing low-quality search results. In our proposed system for this paper we present a new technique to refine Information Retrieval searches to better represent the user’s information need in order to enhance the performance of information retrieval by using different query expansion techniques and apply a linear combinations between them, where the combinations was linearly between two expansion results at one time. Query expansions expand the search query, for example, by finding synonyms and reweighting original terms. They provide significantly more focused, particularized search results than do basic search queries.

**Results:**

The retrieval performance is measured by some variants of MAP (Mean Average Precision) and according to our experimental results, the combination of best results of query expansion is enhanced the retrieved documents and outperforms our baseline by 21.06 %, even it outperforms a previous study by 7.12 %.

**Conclusions:**

We propose several query expansion techniques and their combinations (linearly) to make user queries more cognizable to search engines and to produce higher-quality search results.

## Background

Query expansion techniques are important and widely used for improving the performance of textual information retrieval (IR) systems. These techniques help IR to surmount the issues of vocabulary mismatch because IR focuses on finding documents whose contents match a user query from a large document collection.

Due to the explosive growth of biomedical resources on the web, the amount of stored biomedical information is rapidly growing, and thus effective information retrieval is becoming more difficult [1]. As a consequence, the need for advanced information retrieval systems is all the more pressing. Consider these annual reports which provide the estimated numbers of only new cancer in 2015 and Alzheimer’s disease cases in 2013:

- For new cancer cases and deaths in 2015, as well as current cancer incidence, mortality, and survival statistics and information on cancer symptoms, risk factors, early detection, and treatment the estimated numbers are 1,658,370 new cancer cases diagnosed and 589,430 cancer deaths in the US [2].
- For the United States as a whole, in 2013, the mortality rate for Alzheimer’s disease was 27 deaths per 100,000 people [3].

The procedures of conventional linguistic pre-processing for the documents such as tokenization, steaming, removing stop words and the use of some weighting algorithms e.g. TF-IDF (Term Frequency-Inverse Document Frequency) are not useful enough to achieve results that are related to the user query. Further formulating well-designed queries is difficult for most users, it is necessary to use query expansions to add new related terms to user queries to retrieve relevant information [4].

So, as a result of using information techniques, information retrieval systems can retrieve the required information to index data based on all kinds of predefined searching techniques [5].

In this paper, we built a system for expanding search queries for document retrieval that is relevant, we improved on existing methods for document retrieval by applying different query expansion techniques and combining the results through linear combination. Our proposed approaches achieve good results on the TREC 2006 and 2007 Genomic data-sets, and the experimental results demonstrate a performance improvement when we combined the results of query expansion techniques. Especially the combination between Lavrenko’s relevance model results (Pseudo Relevance Feedback) which is an effective technique for improving retrieval results [6], with the results of query expansion using PubMed Terms [7]. Our results introduce a promising avenue for constructing high performance information retrieval systems in bio-medicine.

The idea behind combination is to obtain performance results much better than that of the individual best results. This is achieved by combining several independent query expansion results and choosing the best results that outperform the baseline.

Our findings, however, do more than outperform the baseline. They even outperform previous studies in the same area that used same data sets [5].

So in brief, we applied our first query expansion approach by using a simple “Most Frequent Terms” technique while tuning different parameter attributes. After that, we applied the second expansion technique to the initial query by using Lavrenko’s relevance model approach by adjusting its different parameter attributes. Subsequently, we also expanded the original query by employing the third expansion technique in this paper using MetaMap Thesaurus. Later, we applied the last expansion technique by expanding the original query using PubMed dictionary from National Library of Medicine (NLM). After each query expansion we evaluated the result scores using a python script that compares to the baseline. Finally, after we obtained the results from the four query expansions, we applied a linear combination which was between two expansion results at one time. We then compared each combination score result with the baseline score.

The remainder of this paper is organized as follows: “Related work” Section provides an overview of related work. “Methods” Section discusses the proposed system and its framework, elaborating on the different query expansion techniques we applied. “Experiments and results” Section outlines the datasets we used, the models we applied, and the results thereof. “Conclusion and future works” Section is the conclusion, and it also touches on avenues for future work.

## Related work

The fast growing character of biomedical information requires good information retrieval systems to provide specific and useful answers in response to complex queries.

Query expansion is one of the major concerns in information retrieval societies. Numerous methods are proposed by researchers to conduct query expansion. Some approaches emphasize on determining expansion terms using unstructured data (Text documents) while the others focus on expansion determination using structured data (Ontologies). Perez-Aguera et al. [8] Compares and combines different approaches for query expansions in unstructured documents. They consider co-occurrence of terms in different documents using Tanimoto, Dice and Cosine coefficients to weigh expansion terms. Also, they analyze the distribution of expansion terms in the top ranked documents and the entire collection of documents using KullbackLiebler Divergence. In [6], Lv et al., published a study about how to select effectively from feedback documents words that are more related to the query topic based on positions of terms in feedback documents. They used a positional relevance model (PRM) to address this problem in a unified, probabilistic way. The results of their experiment on two large web data sets show that the proposed PRM is quite effective and robust and performs significantly better than state-of-the-art relevance model in both document-based feedback and passage-based feedback.

In [9], Alipanah proposed a novel weighting mechanisms for ontology-driven query expansion calling the Basic Expansion Terms (BET) and New Expansion Terms (NET). They considered each individual ontology and user query keywords to determine the Basic Expansion Terms (BET) using a number of semantic measures including Betweenness Measure (BM) and Semantic Similarity Measure (SSM). They propose a Map/Reduce distributed algorithm for calculating all the shortest paths in ontology graph. Rivas et al. in [4] have developed pre-processing techniques of query expansion for retrieving documents in several fields of biomedical articles belonging to the corpus Cystic Fibrosis, a corpus of MEDLINE documents. They conducted experiments showing the different results and benefit of using stemming and stop words in the pre-processing of documents and queries. Their Studies and experiments were conducted to compare the weighting algorithms Okapi BM25 and TF-IDF available in the Lemur tool, concluding that the TF-IDF with TF formula given by BM25 approximation provides superior results. In this paper, we propose multiple query expansion approaches be combined (through Linear combination) to enhance the performance results of the documents retrieved by a query in a scientific documental database.

## Methods

We first started our experiments by indexing a corpus using Indri Toolkit. Indri is a search engine that enables a text search and a full structured query language for text collections of up to 50 million documents (single machine) or 500 million documents (distributed search). Indri is a useful technique whereby using the inference network framework is combined with new theoretical advances in language modeling. It’s an Open Source software and a Part of the Lemur Project and available for Linux, Solaris, Windows and Mac OSX [10–13].

After indexing, we applied basic query searches to the data-set to get baseline results. We used standard parameter attributes and evaluated the results using a Python program attached with Genomics2007 to calculate the result scores with the appropriate gold standard data files available.

There are three levels of retrieval performance measured: passage retrieval, aspect retrieval, and document retrieval. Each of these provides insight into the overall performance for a user trying to answer the given topic questions. Each was measured by some variant of MAP (Mean Average Precision) [14].

**Fig. 1:**
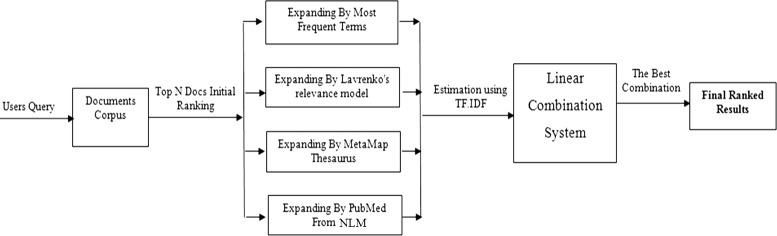
Linear combination of multiple query expansion techniques

Then we applied different Query Expansion approaches by adding new terms to the original queries from different resources. After that and finally we applied a linear combination for the best results we got from query expansion to compare with state-of-the-art (Baseline). In our experiments, we adopted the Indri initial query results as our baseline for later comparison; to be compared with the results of different expansions techniques we applied (before and after) the linear combinations. In the next sections we describe our methods in details. Our model diagram is shown in Fig. 1.

### Indexing

Before indexing the corpus documents, we applied data pre-processing and reformatted the source data to ensure more effective subsequent processing (such as removing HTML tags). We then indexed the collection of documents using Indri toolkit (Library in Java) using the standard index parameters attributes including the default setting memory, index fields, the path of the source collection, and the path of destination folder of the index. First, we pre-processed the documents in order to obtain keywords (relevant words, also named terms) to be used in the query later.

Indexing processes includes:

- Extraction of all the words from each document
- Elimination of the stop-words
- Stemming the remaining words using the porter stemmer, this is the most commonly used [4].

So, While indexing, it is important to take into consideration the use of stemming and stop word lists to reduce related words to their stem, base or root form. This can be achieved by launching affix removal to adapt different derivational or inflectional variants of the same word to a single indexing form and by removing words that do not contain information relevant to the document. Indri Toolkit provides methods in Java for that purpose:

- Krovetz or Porter stemmer as an attributes for setStemmer method
- Include a StopWordsList words as a text file for a setStopwords method

Indexing stemming technique is an effective and good technique to improve MAP (Mean Average Precision) [1]. The results usually vary across weak (Krovetz) and strong (Porter) stemming methods [11, 15, 16], but [4] the results are largely similar. In terms of MAP, Porter is slightly better [4].

### Base line experiment (get initial query results)

After implementing the (initial) queries of 36 topics, the resulting first 1000 relevant documents for each topic were formatted in TREC format. Initial query on the collection of documents was conducted using Indri toolkit with its standard parameter attributes such as setting memory, index fields and the path of the index. Then, we evaluated the results scores using python script. The most frequently applied algorithms for computing the similarity between documents and queries by weighing terms are the TF-IDF and BM25 algorithms. In our experiments we adopted the Indri default algorithm, which is the TF-IDF (Term Frequency and Inverse Document Frequency) algorithm [17]. The main formula for TF-IDF is *tf*_*t,d*_×*idf*_*t*_, in another way and more expanded formula, TF-IDF weight of a term can be calculated as the product of its TF weight and its IDF weight and can be represented as:

1 $$\begin{array}{*{20}l} W_{t,d} = log(1 + tf_{t,d}) \times log(N/df_{t}) \end{array} $$

Where in both formulas, *tf*_*t,d*_ is a t term frequency in the document d, *idf*_*t*_ is the inverse document frequency that contains the term and N is total number of documents [18–20]. Most retrieval systems return a ranked document list in response to a query, where the documents more similar to the query considered by the system are first on the list [4].

### Query expansion and linear combination

Then, after got initial query results, we applied our first query expansion approach by using a simple Most Frequent Terms technique while tuning different parameter attributes the number of terms (Terms No) and evaluating the best results scores and then comparing those results with the baseline results. After that we applied the second expansion technique to the initial query by using Lavrenko’s relevance model approach by adjusting its different parameter attributes to choose the best results scores, which we in turn compared with the baseline results.

We also expanded the original query employing the third expansion technique in this paper using MetaMap Thesaurus. MetaMap is a highly configurable program and a useful tool which is very widely used for the purpose of detecting clinical concepts in text. MetaMap was developed by Dr. Alan (Aronson, 2001) at the National Library of Medicine (NLM). It is an entity recognition software tool used to map biomedical text to the UMLS Metathesaurus or its equivalents [21–24]. In our work here, we used Manual-assigned MetaMap terms and synonyms in creating the query topics, working in two stages, changing the number of terms and then evaluating the best score results and comparing them with the baseline results.

The last expansion technique we used in this paper was by expanding the original query using PubMed dictionary from NLM [7]. We employed Manual-assigned PubMed terms related to the original query terms and then compared the evaluated result scores with the baseline results. PubMed/MEDLINE contains citations and abstracts from approximately 5,516 current biomedicine and health related journals, including works in the fields of medicine, nursing, dentistry, veterinary medicine, health care system and preclinical sciences from the U.S. and over 80 foreign countries; in 39 languages (60 languages for older journals) since 1946 and earlier. There are more than 21 million citations in PubMed/MEDLINE as of November, 2011. About 83 % of them are English citations [7, 25].

Finally, we applied a combination system, here we made a linear combination of the results we got from the four query expansion techniques we applied, where the combination was between two expansion results at one time. We then compared each combination score result with the baseline score results. The Linear Combination (L.C.) formula is:

2 $$\begin{array}{*{20}l} L.C.= \alpha\times Score1+(1- \alpha) \times Score2 \end{array} $$

Where *α* is a weighting attribute, Score1 is the first result to be combined and Score2 is the second results to be combined.

## Experiments and results

Our work in this paper was based on improving the retrieved documents in the corpus. We conducted extensive experiments to compare the evaluated submission results of the query expansion methods by applying different query expansion techniques, then combining the results (two expansion results simultaneously) using linear combination.

Subsequently, we compared the results before and after linear combination with the base line. We also compared our results with previous studies to prove that our model indicates a greater efficiency in retrieving documents.

We used a linear combination to show the effect for combination between each two query expansion results separately, and then compared them. After comparison we found that the combination between Feedback and PubMed Expansion outperformed the baseline by 21.065 %, and outperformed previous study [5] by 7.12 %.

### Tools

We conducted our experiments using Indri Toolkit methods, inside Java library, as our main tool for indexing the corpus and making queries on it. Python programming language was also utilized for measurement and evaluation. The score results and performance was measured by including the gold standard attached with TREC 2007 Genomics [14]. Evaluation was conducted in Command Line prompt.

### Dataset and query topics

The corpus data-set used was the TREC 2006 and 2007 Genomics data-set as full text in HTML format. TREC (Text Retrieval Conference/Competition). The NIST (National Institute of Standards & Technology) administers TREC. We obtained that document collection by a Web crawl of Highwire [26] derived from 49 Journals [14]. We used topics from 2007 as a standard user query in all experiments as a base line query and expanded this query with new terms from different resources as we will describe in detail in the following sections.

#### Expanding query by most frequent terms

The second submission was integrated by conducting some simple relevance feedback techniques based on Most Frequent Terms method. We first used our initial query results as the relevant set and received feedback about the relevancy of results. We then performed subsequent queries based on feedback. Here, the experiments were conducted by tuning different parameter attributes of retrieved documents in two stages:

- The number of retrieved documents was adjusted from 10 to 50, rate of increase was 10, and number of terms was fixed at 10, as the results in Table 1.

**Table 1:** Q.E. using M.F.T., D.N. = (10–50)

MAP	DOC.	PASS.	ASP.	PASS2.
Baseline (Indri)	0.2571	0.0634	0.2008	0.0847
**10**	**0.2729**	**0.0680**	**0.1813**	**0.0880**
20	0.2696	0.0733	0.1960	0.0932
30	0.2688	0.0750	0.1978	0.0949
40	0.2532	0.0721	0.1928	0.0900
50	0.2532	0.0721	0.1928	0.0900
- The number of terms (Terms No) was varied 5 to 30, with an increasing rate of 5 and number of retrieved documents was fixed at 10. as the results show in Table 2.

**Table 2:** Q.E. using M.F.T., T.N. = (5–30)

MAP	DOC.	PASS.	ASP.	PASS2.
Baseline (Indri)	0.2571	0.0634	0.2008	0.0847
5	0.2549	0.0681	0.1867	0.0826
10	0.2574	0.0681	0.1782	0.0870
15	0.2707	0.0711	0.1788	0.0929
**20**	**0.2720**	**0.0716**	**0.1767**	**0.0939**
25	0.2658	0.0670	0.1703	0.0898
30	0.2536	0.0645	0.1509	0.0864

Under this approach, we defined term frequencies in the documents to be the high frequencies of the terms for each query, as relevant terms to the query and then added those terms to the new query. We conducted the new query with added terms. Upon securing the results, we measured and evaluated the scores by comparing with the baseline scores. The highest results are indicated in bold, below. See Tables 1 and 2. Note, in the result tables, Doc. stands for documents, Pass. for Passage, Asp. for Aspect, Pass2. for Passage2, Q.E. for Query Expansion, M.F.T. for Most Frequent Terms, D.N. for Number of Documents and T.N. for Number of Terms.

#### Expanding query using Lavrenko’s relevance model

Pseudo-relevance feedback is one kind of query expansion technique. It begins with an initial query, implements some processing with the initial results, and then returns a list of expansion terms. To get the results of the expanded query the original query is then expanded with the new terms and is executed again. Indri’s pseudo-relevance feedback mechanism is an adaptation of Lavrenko’s relevance model [27]. We implemented it using Indri toolkit [17]. We conducted this experiment in command line prompt with Pseudo-relevance feedback parameters and their attributes, the parameters are <*trecFormat*>, <*runID*>, <*index*>, <*resultFormat*>, <*count*>, <*fbDocs*>, <*fbOrigWeight*>, <*fbTerms*>.

We set <*trecFormat*> attribute to ‘true’, in order to achieve the Trec scorable output. <*runID*> parameter is the name of our submission in this experiment. In the parameter <*index*> here we assigned the path of the index. To produce the results in Trec format we assign ‘trec’ for <*resultFormat*> parameter. The parameter <*count*> was set to 1000 to get the results of 1000 documents for each query topic. We conducted the experiment in three stages by tuning the remaining three parameter attributes as the following:

- Feedback document number <*fbDocs*>: is the number of documents used for feedback. By adjusting different values of <*fbDocs*>=(5−30), with an increasing rate is 5, with fixed value of Feedback Terms number parameter <*fbTerms*>=10 and fixed values of Feedback weight <*fbOrigWeight*>=0.5. After conducting many experiments with the different attributes for the number of documents, we measured and evaluated the result scores as in Table 3.

**Table 3:** Q.E. using Lavrenko’s relevance model, feedback D.N. =(5−30)

MAP	DOC.	PASS.	ASP.	PASS2.
Baseline (Indri)	0.2571	0.0634	0.2008	0.0847
**5**	**0.2866**	**0.0621**	**0.1806**	**0.0911**
10	0.2796	0.0641	0.1910	0.0923
15	0.2791	0.0632	0.1866	0.0903
20	0.2778	0.0653	0.1842	0.0920
25	0.2747	0.0656	0.1893	0.0930
30	0.2761	0.0639	0.1908	0.0927
- Feedback original weight <*fbOrigWeight*>: is a floating point value which can be tuned from 0.1 to 0.9 for specifying the original query weight in the expanded query. By adjusting different value of <*fbOrigWeight*>=(0.1−0.9), where increasing rate is 0.1, with fixed values of Feedback Terms number <*fbTerms*>=10 and fixed value of Feedback Document number <*fbDocs*>=5. After conducting many experiments with the different attributes for query weight, we evaluated the results using python script, see Table 4.

**Table 4:** Q.E. using Lavrenko’s relevance model, feedback weight =(0.1−0.9)

MAP	DOC.	PASS.	ASP.	PASS2.
Baseline (Indri)	0.2571	0.0634	0.2008	0.0847
0.1	0.2591	0.0523	0.1643	0.0807
0.2	0.2591	0.0523	0.1643	0.0807
0.3	0.2724	0.0561	0.1693	0.0838
0.4	0.2791	0.0591	0.1760	0.0878
0.5	0.2866	0.0621	0.1806	0.0911
0.6	0.2943	0.0655	0.1862	0.0941
**0.7**	**0.2974**	**0.0688**	**0.1922**	**0.0965**
0.8	0.2931	0.0707	0.1990	0.0982
0.9	0.2836	0.0712	0.2031	0.0972
- Feedback terms number <*fbTerms*>: is the number of terms used for feedback, by adjusting different values of <*fbTerms*>=(10−60), where the increasing rate is 10, with fixed value of Feedback document number parameter <*fbDocs*>=5 and fixed value of Feedback weight parameter <*fbOrigWeight*>=0.5, then after conducting several experiments with varying attributes for the number of terms, the results after evaluation was obtained and is shown in Table 5.

**Table 5:** Q.E. using Lavrenko’s relevance model, feedback T.N. =(10−60)

MAP	DOC.	PASS.	ASP.	PASS2.
Baseline (Indri)	0.2571	0.0634	0.2008	0.0847
10	0.2866	0.0621	0.1806	0.0911
20	0.2938	0.0645	0.1894	0.0919
30	0.2973	0.0667	0.1945	0.0953
40	0.2980	0.0669	0.1942	0.0948
**50**	**0.2985**	**0.0673**	**0.1920**	**0.0944**
60	0.2982	0.0675	0.1932	0.0945

There were three parameters in the Lavrenko’s relevance model parameter file that required tuning, (<*fbDocs*>,<*fbOrigWeight*> and <*fbTerms*>). We formatted these attributes to select the best results. We put the best evaluated result scores in bold font, which facilitates comparison with the baseline scores.

#### Expanding queries using MetaMap thesaurus

We expanded the original queries in MetaMap by using an online MetaMap tool called Interactive MetaMap [24]. We expanded the original query using MetaMap Thesaurus by manually assigning MetaMap terms and synonyms related to the original query terms. After submitting the query topics (query topics without stop words and punctuations) to MetaMap, we obtained MetaMap candidate texts that related to the original query topics. As an example, the Meta candidate texts related to the term (NF kappaB) in topic 234 are described as *[Amino Acid, Peptide, or Protein, Immunologic Factor]*.

We implemented java code to extract a number of frequent terms for each query topic from that MetaMap texts and repeated this operation 36 times because we have 36 topics (from 200 to 235). The extraction of most frequent terms was in two steps:

- Unordered term numbers.
- 3 term numbers (minimum term numbers).

Unordered term numbers means that the number of extracted most frequent terms from MetaMap candidate texts is not the same for each query topic because some query topics are expanded to 10 terms and some to 8 terms. The minimum expansion had only 3 terms and is labeled as topic 21 in Table 6 below. Table 6 illustrates the query topics that expanded to less than 10 terms.

**Table 6:** Unordered terms numbers

Query topics	T.N. in M.Q.E.
6	8
7	7
9	7
11	5
12	6
13	8
14	5
18	6
20	8
21	3
22	5
23	8
26	8
28	8
29	7
30	6
31	7
35	9

As we mentioned before, we have 36 topics. In other words, the remaining topics, none of which appear in Table 6, expanded with 10 or more MetaMap terms. Note, M.Q.E. stands for MetaMap Query Expansion.

The second step of extracting most frequent terms was to extract only 3 terms (the minimum terms number from step 1) for all topics. We executed the query again after adding the new MetaMap terms of the two steps (unordered terms number and 3 terms number) to the query topics to get the results and then evaluated the result scores to compare with the baseline submission. Table 7, shows the results. The values of MAP (Mean Average Precision) in Table 7 indicated that it didn’t outperform the baseline values; in fact, it didn’t even reach the baseline. However, after linearly combining the different query expansion technique results, we noticed an appreciable difference.

**Table 7:** Q.E. using MetaMap thesaurus

MAP	DOC.	PASS.	ASP.	PASS2.
Baseline (Indri)	0.2571	0.0634	0.2008	0.0847
M.Q.E. T.N. = 3	0.1611	0.0391	0.1419	0.0555
Unordered	0.1554	0.0393	0.1332	0.0542

#### Expanding queries using PubMed dictionary from NLM

Here we expanded the original queries by PubMed online search dictionary [28].

First, we determined PubMed terms and their synonyms by implementing a manual search for each query one by one.

After obtaining the PubMed resulting documents that were related to each query topic, we just copied the abstracts of all documents related to one query topic to a text file; each query topic in a separate text file.

Java programming code was employed to obtain the Most Frequent Terms for each query file in two steps, first with number of Terms =5 then number of Terms =10. After which, the query was subsequently executed, adding new PubMed terms following the same two step process. The results are shown in Table 8. Note, P.Q.E. stands for PubMed Query Expansion.

**Table 8:** Expanding query by PubMed

MAP	DOC.	PASS.	ASP.	PASS2.
Baseline (Indri)	0.2571	0.0634	0.2008	0.0847
P.Q.E T.N. = 10	0.2014	0.0446	0.1522	0.0614
T.N. = 5	0.2199	0.0499	0.1701	0.0709

As is clear in Table 8, the values of MAP (Mean Average Precision) also didn’t outperform the baseline values. The values, however, were more precise than the MetaMap results copied in Table 7. Later, upon making a linear combination between different query expansion technique results we saw higher-quality search results.

#### Linear combinations and comparison between results

We used java programming code for conducting the linear combination experiment to simultaneously combine two different result scores. According to equation (2), in the experiments, *α* value was tuned with values from 0.1, 0.2 to 0.9 for each execution. All combined results were evaluated using python script. After which we chose the best value (highest) for evaluation.

The results, copied in Tables 9, 10, 11, 12, 13 and 14 are striking and apparent. Note, in the result tables, T.N. stands for Number of Terms, D.N. for Number of documents and U.O.T. stands for number of Unordered Terms. The highest combination results are highlighted in bold for easy reference vis-a-vis the baseline results.

**Table 9:** Feedback & MetaMap combination

MetaMap	Feedback	DOC.	PASS.	ASP.	PASS2.
Attributes	Attributes	MAP	MAP	MAP	MAP
Baseline (Indri)		0.2571	0.0634	0.2008	0.0847
T. N. = 3	D.N. = 5	0.2811	0.0638	0.1903	0.0890
U. O. T.		0.2776	0.0629	0.1904	0.0874
**T.N.** =**3**	**T.N.** =**40**	**0.2901**	**0.0662**	**0.2012**	**0.0907**
U.O.T.		0.2843	0.0643	0.1981	0.0883
T. N. = 3	Weight = 0.7	0.2824	0.0664	0.1977	0.0920
U. O. T.		0.2780	0.0645	0.1974	0.0890

**Table 10:** Feedback & PubMed combination

PubMed	Feedback	DOC.	PASS.	ASP.	PASS2.
Attributes	Attributes	MAP	MAP	MAP	MAP
Baseline (Indri)		0.2571	0.0634	0.2008	0.0847
T.N. = 5	D.N. = 5	0.2993	0.0683	0.2002	0.0959
T.N. = 10		0.2961	0.0669	0.1909	0.0942
T.N. = 5	**T.N.** =**40**	0.3064	0.0706	0.2059	0.0983
**T.N.** =**10**		**0.3113**	**0.0703**	**0.1999**	**0.0973**
T.N. = 5	Weight = 0.7	0.3044	0.0708	0.2018	0.0988
T.N. = 10		0.3087	0.0704	0.2035	0.0975

**Table 11:** Feedback & M.F.T. combination

M.F.T.	Feedback	DOC.	PASS.	ASP.	PASS2.
Attributes	Attributes	MAP	MAP	MAP	MAP
Baseline (Indri)		0.2571	0.0634	0.2008	0.0847
T.N. = 20	D.N. = 5	0.2929	0.0708	0.1903	0.0977
	**T.N.** =**40**	**0.3001**	**0.0711**	**0.1984**	**0.0983**
	Weight = 0.7	0.2997	0.0725	0.1962	0.1000

**Table 12:** M.F.T. & MetaMap combination

M.F.T.	MetaMap	DOC.	PASS.	ASP.	PASS2.
Attributes	Attributes	MAP	MAP	MAP	MAP
Baseline (Indri)		0.2571	0.0634	0.2008	0.0847
T.N. = 20	**T.N.** =**3**	**0.2755**	**0.0692**	**0.1887**	**0.0909**
	U.O.T.	0.2687	0.0664	0.1857	0.0874

**Table 13:** M.F.T. & PubMed combination

M.F.T.	PubMed	DOC.	PASS.	ASP.	PASS2.
Attributes	Attributes	MAP	MAP	MAP	MAP
Baseline (Indri)		0.2571	0.0634	0.2008	0.0847
T.N. = 20	**T.N. = 5**	**0.2886**	**0.0739**	**0.1955**	**0.0979**
	T.N. = 10	0.2881	0.0739	0.1977	0.0982

**Table 14:** PubMed & MetaMap combination

PubMed	MetaMap	DOC.	PASS.	ASP.	PASS2.
Attributes	Attributes	MAP	MAP	MAP	MAP
Baseline (Indri)		0.2571	0.0634	0.2008	0.0847
T.N. = 5	**T.N.** =**3**	**0.2484**	**0.0589**	**0.1994**	**0.0799**
T.N. = 10		0.2454	0.0573	0.1827	0.0754
T.N. = 5	U.O.T.	0.2407	0.0563	0.1893	0.0761
T.N. = 10		0.2337	0.0546	0.1756	0.0729

#### Results and discussion

**Fig. 2:**
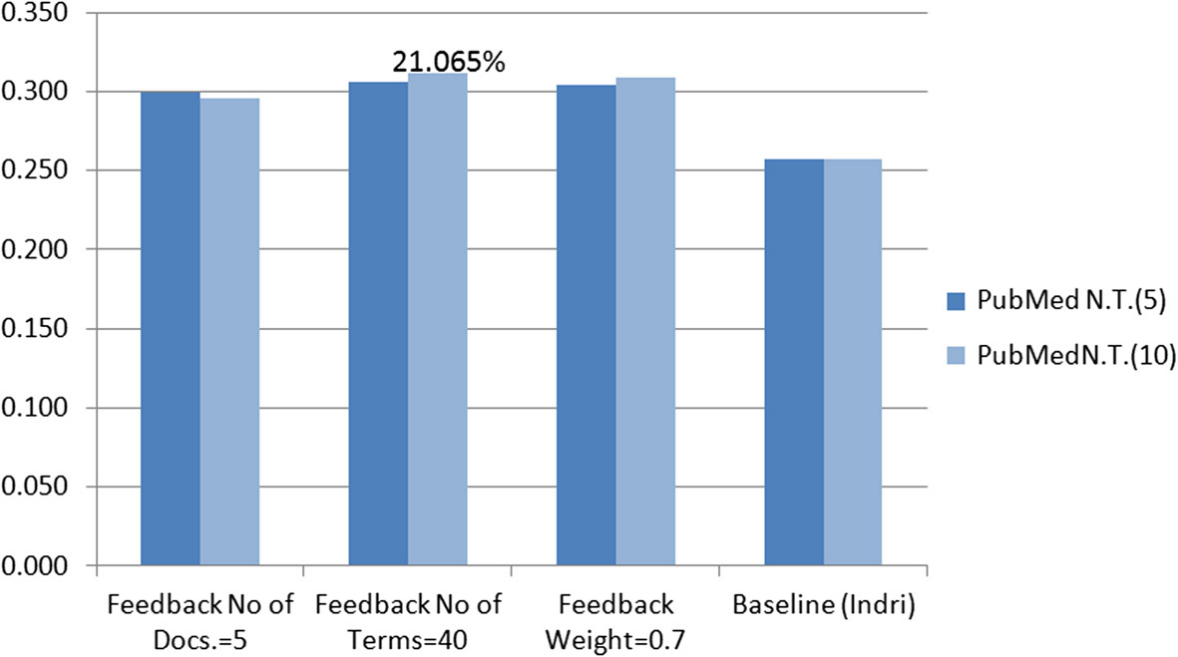
Combination of feedback and PubMed Q.E

We start discussion with the best results of linear combination between Feedback and PubMed query expansions, see Table 10, where the Mean Average Precision (MAP) of documents in this combination between Feedback query expansion and PubMed query expansion with their best attributes for the parameters (No. of Terms =40 and 10 respectively) outperformed the baseline (Indri) by 21.065 %, which is a marked improvement of previous studies by 7.12 % which using the same datasets [5], see Fig. 2. The combination between Feedback and Most Frequents Terms query expansions with the best attributes of their parameters works very well and indicate the advantage in MAP of documents, as it shown in Table 11.

**Fig. 3:**
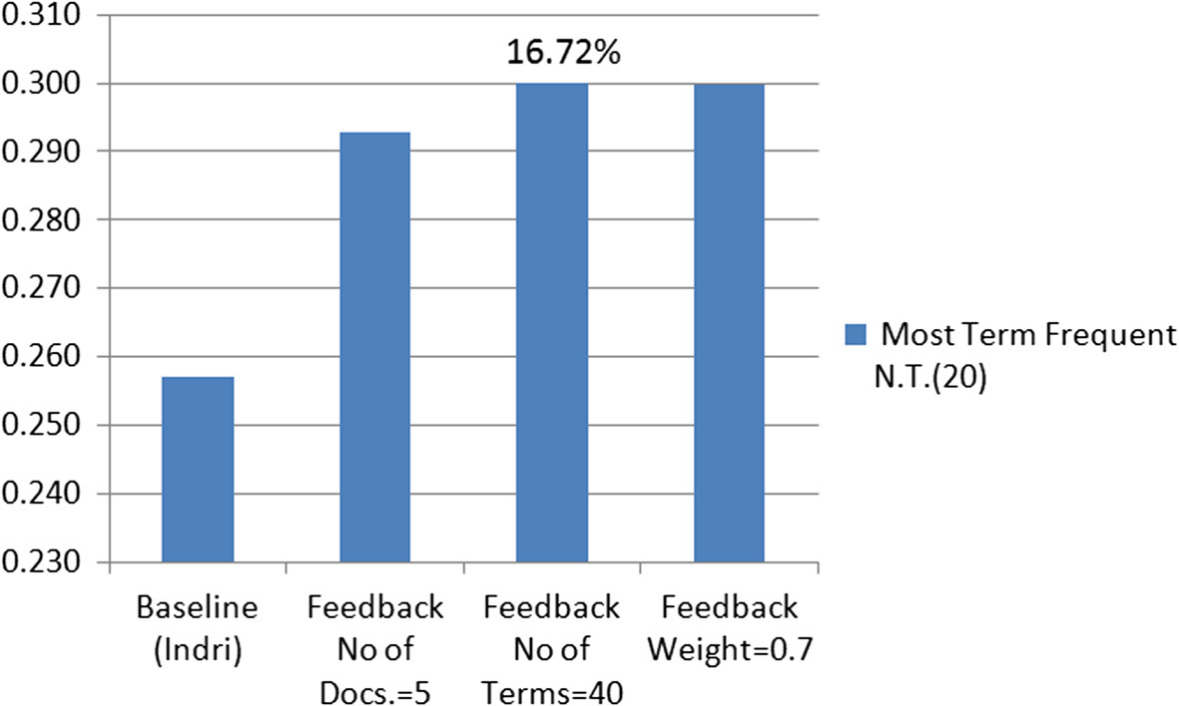
Combination of feedback and M.F.T

**Fig. 4:**
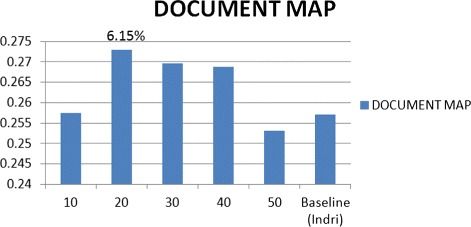
Q.E. using M.F.T. with D.N. parameter

**Fig. 5:**
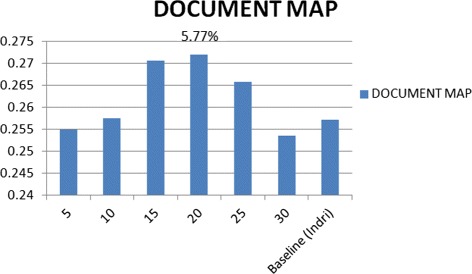
Q.E. using M.F.T. with T.N. parameter

Where the linear combination between best results of these two query expansions (Feedback and M.F.T.) with their best attributes for the expansion parameters (No. of Terms = 40 and 20 respectively) outperformed the baseline (Indri) by 16.72 %, which outperforms the previous study by 3.27 % using the same datasets that we used [5]. See Fig. 3. The results of expanding the query by the method of M.F.T., the MAP outperformed the baseline (Indri) by 6.15 and 5.77 % with the best attributes of parameters Doc. No. & Terms No. respectively, See the flowchart in Figs. 4 and 5.

**Fig. 6:**
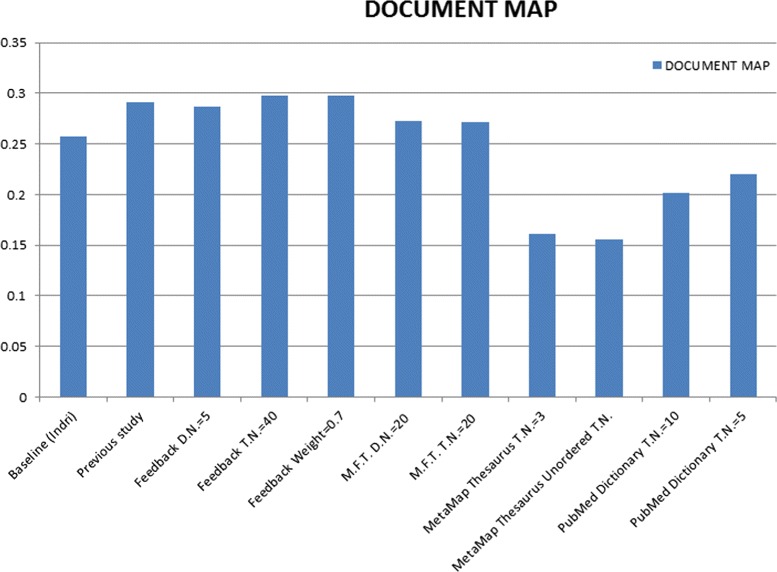
Q.E. individually with their parameters

For a general comparison between all query expansion techniques and the baseline used in this paper and the previous study, see Table 15 and Fig. 6, best result in bold.

**Table 15:** Best results for different Q.E.

Expansions with different parameters	DOC. MAP
Baseline (Indri)	0.2571
Previous study	0.2906
Feedback D.N. = 5	0.2866
**Feedback T.N.** =**40**	**0.2980**
Feedback Weight = 0.7	0.2974
M.F.T. D.N. = 20	0.2729
M.F.T. T.N. = 20	0.2720
MetaMap Thesaurus T.N. = 3	0.1611
MetaMap Thesaurus Unordered T.N.	0.1554
PubMed Dictionary T.N. = 10	0.2014
PubMed Dictionary T.N. = 5	0.2199

**Fig. 7:**
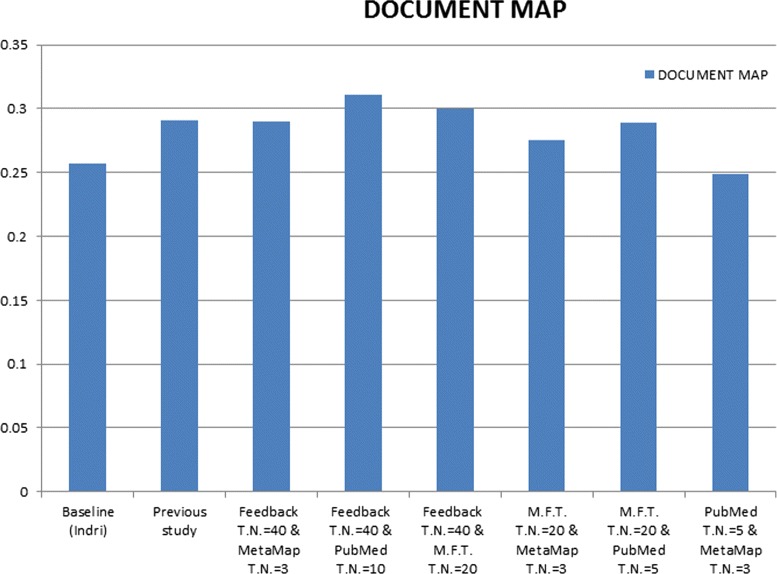
Different Q.E. combinations individually

The comparison between all evaluated results of combinations for results scores of query expansion methods and baseline we used in this paper in addition to previous study [5], see Table 16 and Fig. 7, the best result is highlighted in bold.

**Table 16:** Best results for Q.E. combinations

Best combinations	DOC. MAP
Baseline (Indri)	0.2571
Previous study	0.2906
Feedback T.N. = 40 & MetaMap T.N. = 3	0.2901
**Feedback T.N.** =**40 & PubMed T.N.** =**10**	**0.3113**
Feedback T.N. = 40 & M.F.T. T.N. = 20	0.3001
M.F.T. T.N. = 20 & MetaMap T.N. = 3	0.2755
M.F.T. T.N. = 20 & PubMed T.N. = 5	0.2886
PubMed T.N. = 5 & MetaMap T.N. = 3	0.2484

## Conclusion and future works

We present a new technique to refine Information Retrieval searches to better represent the user’s intended search. First, we started our experiments by indexing a corpus using Indri Toolkit, which was used to obtain the baseline results (we adopted the initial Indri query results as Baseline) with its standard parameter attributes, and then evaluated its results by using a python script attached with TREC 2007 Genomics as we described in the experiments section. Second, we applied four query expansion methods by using Most Frequent Terms technique, Lavrenko’s relevance model (Pseudo Relevance Feedback approach), expanded using MetaMap Thesaurus and expanding the original query using PubMed dictionary from NLM, by tuning the different parameters and then compared the evaluated results scores with the Base Line submission. Third, we applied a linear combination for each two expanding approaches, after choosing the best combinations and comparing them with the baseline, we concluded that our results were enhanced and outperformed our Base Line (Indri) by 21.065 %, and further outperformed the previous study [5] by 7.12 %.

Our future work is to expand the original query by using Wikipedia thesaurus and WordNet online search tool, by adding new terms to the query topics, and then combining all query results using an alternate method, such as CombMNZ combination algorithm, in order to apply some ranking techniques for the results and improve the performance.

## Abbreviations

Asp., aspect; Doc., documents; D.N., number of documents; L.C., linear combination; MAP, mean average precision; M.F.T., most frequent terms; M.Q.E., metamap query expansion; NIST, National Institute of Standards & Technology; NLM, National Library of Medicine; Pass., passage; Pass2., passage2; P.Q.E., pubmed query expansion; Q.E. query expansion; TF-IDF, term frequency-inverse document frequency; TREC, text retrieval conference/competition; T.N., number of terms; UMLS, unified medical language system; U.O.T., number of unordered terms
